# 2496. Hepatitis C Virus Among Inpatients in the Direct-acting Antiviral Era: Prevalence, Linkage to Care, Treatment Disparities, and Comorbidity Burden

**DOI:** 10.1093/ofid/ofad500.2114

**Published:** 2023-11-27

**Authors:** Nicholas Forrister, Austin Nguyen, Ricardo A Franco, David Redden

**Affiliations:** University of Alabama at Birmingham, Birmingham, Alabama; University of Alabama at Birmingham, Birmingham, Alabama; University of Alabama at Birmingham, Birmingham, Alabama; University of Alabama at Birmingham, Birmingham, Alabama

## Abstract

**Background:**

The advent of direct-acting antivirals (DAAs) calls for optimized hepatitis C virus (HCV) screening, linkage to care (LTC), access to cure, and mitigation of disease burden among health systems. We assessed HCV burden and care gaps among inpatients in a large volume, tertiary care setting during the DAA era.

**Methods:**

This is a retrospective cohort analysis of inpatients admitted at least once to medicine services from 2014 to 2021. We used analytic software to query electronic health records. We assessed demographics, comorbidities, HCV diagnosis, LTC, and treatment occurring at any point during the study period. Odds ratios (OR) were utilized to assess differences in key comparator groups.

**Results:**

Over the sampling period, 79793 unique patients were evaluated. The care cascade flowchart is displayed in Figure 1. Overall, 44136 were screened for HCV (55%). Of those screened, 3475 (8%) had a positive viral load. Viremic patients were more likely males (OR 2.04; CI 1.89-1.92), patients with mental health disorders (OR 1.29; CI 1.02-1.38), or opioid use disorder (OR 7.3; CI 5.81-9.21). These same groups were also more likely to be screened. Among viremic patients 1641 (47%) attended treatment clinics. Those with mental health disorders (OR 1.28; CI 1.12-1.46) and alcohol use disorder (OR 3.02; CI 2.5-3.65) were more likely to attend clinics. Those less likely to establish care were Millennials (OR 0.22; CI 0.18-0.27), Generation-X (OR 0.44; CI 0.37-0.52), and patients with opioid use disorder (OR 0.60; CI 0.41-0.87). A total of 950 (58%) patients were prescribed DAAs. Millennials (OR 0.44; CI 0.32-0.61), Generation-X (OR 0.61; CI 0.48-0.78), and those from rural areas (OR 0.62; CI 0.41-0.94) were less likely begin DAA. Those with HIV co-infection (OR 1.83; CI 1.23-2.74) and cirrhosis (OR 1.63; 1.27-2.09) were more likely to start DAA.

**Figure:**
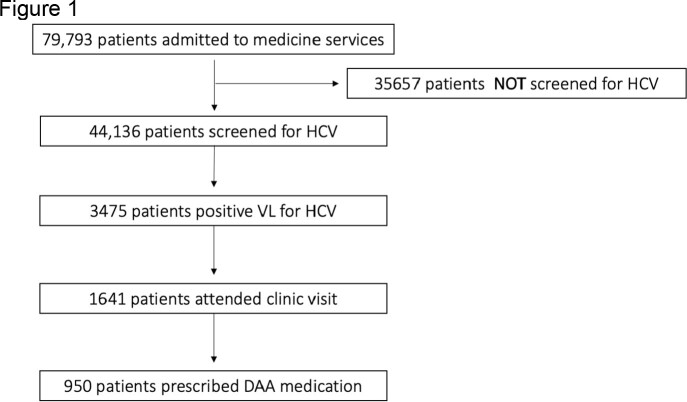

Flow chart examining HCV care cascade of inpatients admitted to at least once to medicine services at the University of Alabama at Birmingham hospital from January 1 2014 to December 31 2021. T flow chart depicts number of inpatients who underwent HCV screening, confirmation of viremia, clinic attendance, and treatment initiation at any point during the study period.

**Table 1:**
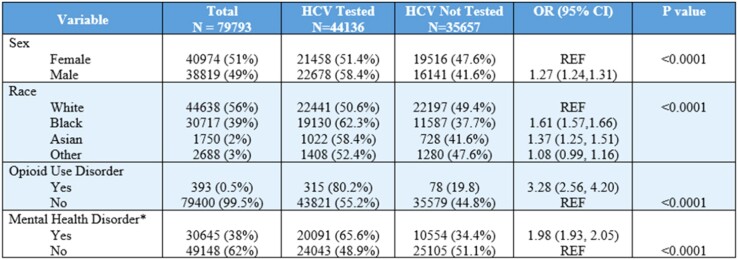
Baseline characteristics of inpatients admitted to medicine services from 2014 to 2021 and status of testing for Hepatitis C virus infection. Mean age is 62 with standard deviation of 17.8 years. *Diagnosis based on ICD-10 diagnosis codes in electronic medical records.

**Conclusion:**

In this large longitudinal analysis of inpatients cared for at a tertiary safety net hospital, a high proportion of individuals underwent HCV screening, and infection was highly prevalent. Care disparities remain and further optimization of HCV screening, LTC, and treatment will need to target younger populations, those with opioid use disorder, and living in rural areas.

**Disclosures:**

**Ricardo A. Franco, MD**, Abbvie: Grant/Research Support|Gilead: Advisor/Consultant|Gilead: Grant/Research Support|Merck: Grant/Research Support|Theratechnologies: Advisor/Consultant

